# Evaluation of Mild Cognitive Impairment through Perientorhinal/Hippocampal Imaging and Comprehensive Neuropsychological and Psychophysical Assessment

**DOI:** 10.3390/brainsci14070697

**Published:** 2024-07-12

**Authors:** Sara Invitto, Paolo Boscolo-Rizzo, Giacomo Spinato, Giuseppe Trinchera, Giuseppe Accogli, Vincenzo Ciccarese, Luca Saba, Marcella Caggiula, Gaetano Barbagallo, Alfredo Pauciulo, Marina de Tommaso

**Affiliations:** 1 Laboratory on Cognitive and Psychophysiological Olfactory Processes, Department of Biological and Environmental Science and Technologies, University of Salento, 73100 Lecce, Italy; 2 Department of Medical, Surgical, and Health Sciences, Section of Otolaryngology, University of Trieste, 34127 Trieste, Italy; pboscolorizzo@yahoo.it; 3 Neuroscience Department, University of Padova, 35122 Padova, Italy; giacomo.spinato@unipd.it; 4 Dipartimento di Scienze Mediche di Base, Neuroscienze e Organi di Senso, University of Aldo Moro Bari, 70121 Bari, Italy; giuseppe.trinchera89@gmail.com (G.T.); marina.detommaso@uniba.it (M.d.T.); 5 Scientific Institute I.R.C.C.S. Eugenio Medea, Via D. L. Monza 20, 23842 Bosisio Parini, Lecco, Italy; giuseppe.accogli@lanostrafamiglia.it; 6 Istituto Santa Chiara, 73100 Lecce, Italy; ciccareseenzo@libero.it; 7 Department of Medical Sciences and Public Health, University of Cagliari, 09100 Cagliari, Italy; lucasaba@unica.it; 8 Division of Neurology, Vito Fazzi Hospital, 73100 Lecce, Italy; m_caggiula@hotmail.com (M.C.); gaebarbagallo@gmail.com (G.B.); 9 Division of Neuroradiology, Vito Fazzi Hospital, 73100 Lecce, Italy; alfredopauciulo@gmail.com

**Keywords:** olfactory perception, mild cognitive impairment, enthorinal cortex, anxiety, neurodegenerative processes, biomarkers, ERICA

## Abstract

Mild cognitive impairment (MCI) is a significant concern as it is a risk factor for AD progression, and early detection is vital in order to delay dementia onset and enable potential therapeutic interventions. Olfactory impairment is recognized as a predictive biomarker in neurodegenerative processes. The aims of this study were to explore the degree of entorhinal cortical atrophy (ERICA) and the severity of MCI symptoms; to analyze magnetic resonance imaging (MRI) results for the entorhinal cortex, parahippocampal gyrus, peri entorhinal cortex, and the cerebellar tentorium; and to perform a comprehensive neuropsychological and psychophysical assessment. The main results highlighted that in our sample—multidomain amnesic MCI patients with hyposmic symptomatology—we found that ERICA scores were associated with the severity of anxiety symptomatology. One possible hypothesis to explain this observation is that anxiety may contribute to neurodegenerative processes by inducing chronic stress and inflammation. Future research should consider the longitudinal development of neuropsychological scores, anxiety disorders, and brain atrophy to determine their potential predictive value for MCI progression. These findings suggest the importance of psychological factors in MCI progression and the utility of neuropsychological assessment alongside neuroimaging techniques for early detection and follow-up in MCI patients.

## 1. Introduction

Mild cognitive impairment (MCI) can represent an intermediate stage between normal cognitive aging and dementia, notably Alzheimer’s Disease (AD) [1]. Individuals with MCI typically display cognitive deficits that exceed those expected for their age and education level, but these impairments do not significantly hinder their daily activities [2]. Despite this, MCI is of significant concern as it has been identified as a risk factor for progression towards AD [3]. Therefore, early detection of MCI is crucial in order to delay the onset of dementia and to allow the implementation of potential therapeutic interventions [4]. Some studies on MCI subjects have shown that not all subjects develop dementia, but only a percentage varying from 12 to 30% per year. Furthermore, patients can remain stable over time from a cognitive point of view, or even improve [5].

The scientific community’s efforts are currently aimed towards identifying those at the most significant risk of conversion. In particular, research is currently aimed, on the one hand, at identifying biological and neurophysiological markers (e.g., cerebrospinal fluid and morphological, electrophysiological, and functional imaging) that could be predictive of conversion, and, on the other, at identifying neuropsychological tools that are increasingly sensitive to the cognitive deficits that characterize subjects with MCI destined to evolve into dementia [6].

Moving away from the identification and international classification of MCI, we know that mild cognitive decline can be classified as a deficit in cognitive function at its onset, and it can be identified as one of four subtypes [7]:

(1) Single domain amnesic MCI: this is a rare form that only involves memory and correlates with the evolution of Alzheimer’s disease;
(2) Multiple domain amnesic MCI represents the prevalent form, in which memory is compromised together with one or more different cognitive domains;
(3) Non-amnesic single domain MCI exclusively involves the cognitive domain, except memory;
(4) Non-amnesic multiple domain MCI, in which two or more cognitive domains are involved, except memory.

From some of the research in the literature, it has emerged that the type of dementia that the person diagnosed with MCI will develop depends on the type of cognitive domain that deteriorates during the initial phase. For example, we can see that patients with deterioration only and exclusively in the memory domain will almost certainly develop Alzheimer’s disease; patients with deterioration of multiple cognitive domains will develop vascular dementia or Alzheimer’s; and finally, those who present deterioration of only one cognitive domain other than memory will more likely develop frontotemporal dementia, Lewy body dementia, vascular dementia, Alzheimer’s disease, or progressive aphasia [8,9].

Most authors have considered memory tests to be the best predictor of the development of AD in healthy elderly subjects and patients classified as having MCI—in subjects with MCI, the predictive memory disorder appears more selective and is represented by different recalls, independent of the test mode, stimulus (visual or verbal), and the structure of the memorization (word list or story).

In subjects in the preclinical phase of AD, episodic, semantic, and working memory tests appear to represent the predictors.

Furthermore, many studies report a semantic memory deficit (semantic fluency) in a high percentage of patients with MCI who develop AD. In contrast, others think it is due to damage to the perirhinal/entorhinal cortex.

Returning to the peculiarities of this disorder, we must mention the impairment of short- and long-term memory; at least one cognitive alteration (between aphasia, apraxia, and agnosia or alteration of executive function); and the impairment of adaptive behavior. Related to cognitive deterioration in dementia, we find ‘Pseudo’ Dementia with symptoms such as memory loss, difficulty concentrating, language disorder, loss of appetite, and other behavioral symptoms that may be present in the depressive form or in other psychiatric syndromes that are mistaken for forms of dementia. It is essential to recognize that depression and dementia can share common symptoms, and to accurately differentiate between patients with depression who are suspected of having dementia [10].

Regarding the diagnosis, however, accurate discrimination of the typical aspects of dementia, typical of depression and areas of comorbidity, is necessary.

In regard to the diagnosis, it is necessary, through genetic tests and techniques based on neuroimaging, to diversify from dementia and give high importance to the neuropsychological evaluation, as the latter allows us to discern the early signs of a syndrome and define the subtypes depending on the prevalence or otherwise of memory disorders and other concomitant pathologies.

The amnesic type appears to be the one that most predisposes the individual to developing dementia, and other factors, such as the comorbidity of depression, seem to indicate a significant impact on the evolution of dementia.

Many recent studies indicate how the compromised olfactory response can be considered a biomarker of the development of neurodegenerative pathology [6,11,12,13]. Due to olfactory processing’s reliance on the mesial temporal areas, which are among the first to exhibit neuropathological changes in prodromal AD, olfactory perception reflects the underlying pathology in the earliest stages of this disease [14]. In particular, one of our previous studies indicates how the electrophysiological responses in MCI are compromised in the early components of chemosensory event-related potentials (CSERPs) and olfactory event-related potentials (OERPs), and are compensatory in the slower components [12]. This indicates that, even if anosmia cannot be diagnosed psychophysically, the electrophysiological and psychophysiological traits can show precisely how the impairment is visible with cortical imaging. The cortical site that should elicit the response of the OERPs is the entorhinal and perirhinal cortex [15,16,17].

The entorhinal cortex incorporates several parts of the olfactory cortex and sends and receives information to and from the hippocampus, the limbic system’s main structure [18]. Nonetheless, this connection is hypothesized to stimulate olfactory memory [19].

Any anomalies affecting the entorhinal cortex are associated with pathologies of different natures to AD [13,20]. Thus, the accumulation of the mutated Tau protein and the neurofibrillary clusters it produces tends to occur mainly in this area.

Recent functional magnetic resonance imaging (fMRI) studies identified the entorhinal area as the gateway to AD [21]. Entorhinal deterioration causes cognitive disorders that gradually reduce hippocampus volume, which is a typical condition of patients with AD.

On the other hand, psychiatric diseases—such as schizophrenia—and mood disorders are associated with impairment of the entorhinal cortex [22]. Anxiety and depression can alter olfactory perception. Depressed individuals, for example, may experience hyposmia, which could be linked to neurobiological changes in brain areas related to mood. A recent electrophysiological study shows us how the latencies of slow components elicited by trigeminal stimuli are directly proportional to anxious states in healthy subjects; this further demonstrates how the olfactory pathway is actually strongly implicated in other functional dimensions, both emotional and cognitive [23].

Moreover, the perientorhinal and hippocampal regions are critically involved in memory and spatial navigation [18], functions that are mildly impaired in MCI and strongly impaired in AD. In AD, these areas are among the first to undergo pathological changes such as atrophy, progressively affecting other brain regions [24].

Hippocampal and entorhinal atrophy (ERC) plays an important role in designing biomarkers for patients with “MCI due to AD”. Compared to healthy subjects, hippocampal volumes for AD patients are reduced by 26–27% and ERC volumes by 38–40%; MCI patients show intermediate medial temporal lobe (MTL) and ERC atrophy levels. MCI is known for frontal and temporal GM loss, atrophy in the primary olfactory cortex, and some basal forebrain cholinergic system structures.

As the disease progresses, atrophy advances to the rest of the MTL, where gray matter (GM) loss occurs in the medial temporal gyrus, parahippocampus, parahippocampal and fusiform gyrus, and temporal lobe. Nesteruk et al. found that MTL atrophy discriminates MCI to AD converters from non-converters [25].

Additional limbic structures, including the amygdala, olfactory bulb tract, cingulate gyrus, and thalamus, are affected in AD. As the disease progresses, atrophy spreads to cortical regions. The frontal, parietal, and temporal brain areas undergo volumetric reductions, as do the putamen and the basal forebrain cholinergic system. Atrophy is also found in the primary olfactory cortex and lower-level brain areas, including the cerebellum and brainstem. Structural MRI scans also display white matter hyperintensities (WMHs), indicating demyelination and axonal loss. Compared to healthy subjects, AD patients show greater WMHs, most of which are in the frontal lobe. For patients along the AD spectrum, WMHs are related to hippocampal atrophy, in addition to neuropsychological deterioration and psychiatric disorders. Moreover, neuropsychological assessment is an integral part of the diagnostic process for MCI. It comprehensively evaluates cognitive functions, including memory, attention, executive functions, language, and visuospatial abilities [26]. Starting from these theoretical premises linked to biomarkers in MCI—since we have already studied MCI and the electrophysiological olfactory response (not always finding anosmia or hyposmia, but more often a basic sensorial compensatory process that covers a perceptive olfactory impairment)—the aim of this study was to begin to investigate, in a preliminary way, through a neuroimaging tool with a high spatial resolution (but lower temporal resolution), the entorhinal, perirhinal, and parahippocampal impairment processes of our subjects and whether their levels of impairment/atrophy are connected to behavioral and psychophysical aspects that can be assessed through a complete neuropsychological, psychophysical, and psychological investigation. ERICA score [1] was chosen because the entorhinal cortex and the transentorhinal region are among the first brain structures to show pathological changes in AD, even before such changes appear in the hippocampus [27], and are representative of the olfactory, spatial, and emotional functions.

## 2. Materials and Methods

### 2.1. Subjects

This is a cross-sectional study conducted between January 2022 and September 2022. A total of 48 MCI subjects (mean <range> age, 71 <51–82> years; 24 <50.0%> females) were recruited for the study at the Unit of Neurology, Vito Fazzi Hospital, Lecce, Italy. Inclusion criteria were as follows: (1) age ≥ 60 years; (2) subjective cognitive complaints; (3) objective cognitive impairment in one or more domains, not severe enough to interfere with daily function; (4) preserved general cognitive function; (5) absence of dementia. Exclusion criteria included the following: (1) diagnosis of dementia according to DSM-V criteria; (2) active neurological or psychiatric disorders that could account for cognitive deficits; (3) history of traumatic brain injury, stroke, or other neurological conditions; (4) sensory impairments that could significantly impact cognitive testing; and (5) inadequate proficiency in the Italian language.

Patients with confirmed MCI completed the neuropsychological and psychophysical assessment with the following tests: MMSE (Mini-Mental State Examination) [28], Digit Span [29], Rey Auditory Verbal Learning Test (RAVL) [30], CORSI Block Tapping Test [31], TMT (Trail Making Test) [32], Sniffin’ Sticks Test for olfactory perception [33], Prose Memory: Babcock Story [34], FAB (Frontal Assessment Battery) [35], Visual Attention Test (Attentive Matrices), BAI (Beck Anxiety Inventory), BDI-II (Beck Depression Inventory II), Activities of Daily Living (ADL), Instrumental Activities of Daily Living (IADL), G8 Geriatric Assessment, Mediterranean diet adherence. No assessment was performed in the neurology unit for SARS-CoV-2, despite the fact that this infection can lead to inflammatory and neurodegenerative processes, which partly overlap with MCI symptoms [36].

The patients were evaluated for the neuropsychological and psychophysics assessment in the INSPIRE Laboratory, a laboratory focused on psychophysiological and cognitive processes related to olfaction in the Department of Biological and Environmental Sciences and Technologies, University of Salento. The same patients were evaluated for the neuroradiological assessment in the Neuroradiology Operative Unit of Vito Fazzi Hospital in Lecce. The study was conducted in accordance with the Declaration of Helsinki, and approved by the Ethics Committee of Vito Fazzi Hospital, protocol Verb. N36 (date of approval: 13 May 2016) and addendum n. 74 (date of approval: 22 April 2022). The patients signed an informed consent form before starting the neuropsychological evaluation battery.

### 2.2. Perientorhinal/Hippocampal MRI

**Figure 1:**
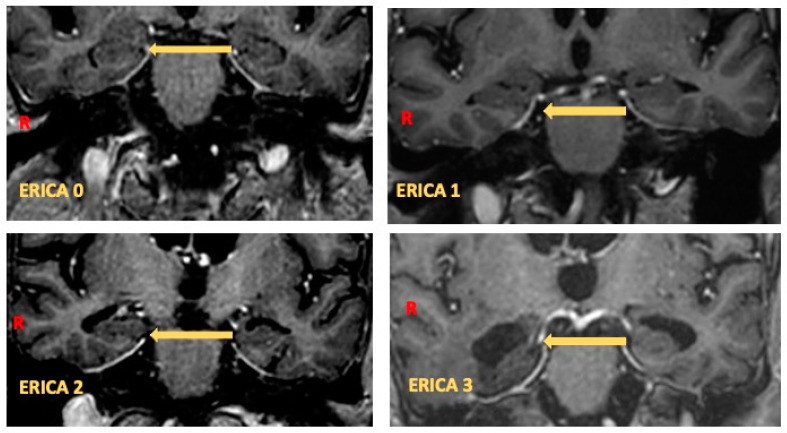
ERICA scores in coronal MRI slice: ERICA score 0, normal volume of entorhinal cortex and parahippocampal gyrus; ERICA score 1, mild atrophy with widening of collateral sulcus; ERICA score 2, moderate atrophy of entorhinal cortex with “tentorial cleft sign”; and ERICA score 3, emphatic atrophy of parahippocampal gyrus with wide cleft between entorhinal cortex and cerebellar tentorium.

All patients with MCI underwent MRI, performed on a 1.5 T scanner according to general brain MR protocol. MRI Images were acquired with a 1.5 T MR scanner imaging unit (Achieva, Philips Healthcare, Best, The Netherlands). Coronal sections aligned to the brainstem with a section thickness of 1 mm were evaluated after software-based multiplanar reconstruction of high-spatial resolution three-dimensional T1-weighted sequences (isotropic three-dimensional gradient echo; voxel size, 1.0 × 1.0 × 1.0 mm; echo time, 3.8 ms; repetition time, 8.3 ms). MRI images were visually inspected by the neuroradiologist to ensure sufficient technical quality and to exclude individuals with neuroradiologic conditions that could potentially interfere with cognitive functioning (e.g., cortical infarction, tumor, subdural hematoma, hydrocephalus). No subject belonged to these clinical categories. Visual evaluation of entorhinal cortex atrophy was performed using a four-point entorhinal cortical atrophy (ERICA) scoring system. Measurements were made by an expert neuroradiologist on coronal images according to a plane parallel to the third ventricle weighted T1. ERICA scores [20] were defined as score 0 for normal volume of the entorhinal cortex and parahippocampal gyrus; ERICA score 1 for mild atrophy with widening of the collateral sulcus; ERICA score 2 for moderate atrophy of the entorhinal cortex with “tentorial cleft sign”; and ERICA score 3 for emphatic atrophy of the parahippocampal gyrus with a wide cleft between the entorhinal cortex and the cerebellar tentorium (see Figure 1).

### 2.3. Statistical Analysis

Descriptive analysis evaluated the neuropsychological and olfactory scores’ mean (m) and standard deviation (sd).

Shapiro–Wilk normality tests were performed on each neuropsychological test to assess the normality of the sample used.

To evaluate gender differences in the group’s psychophysical olfactory characteristics (i.e., the Sniffin’ Sticks Test), a parametric one-way analysis of variance (ANOVA) was performed.

Effect sizes were calculated as eta-squared (η^2^) for analysis of variance (ANOVA) and Cohen’s d for Tukey’s post hoc test. To examine the effect of the degree of cerebral atrophy on neuropsychological test performance, we performed a single one-way nonparametric ANOVA for each test with a nonparametric distribution and a one-way ANOVA for each test with a normal distribution, as well as multiple linear regression (STEPWISE method) considering all the comprehensive neuropsychological assessments.

Moreover, a power analysis was performed to evaluate the specified design.

## 3. Results

Descriptive analysis showed mild impairment in MMSE scores (MMSE m = 24; sd = 4.8) and mild or strong impairment in other cognitive domains (15 Ray W Imm m = 31.33, sd = 8.9; 15 Ray W diff m = 5.97, sd = 3.3; ADL m = 5.4, sd = 0.96; BAI m = 12.3, sd = 10.9; BDI m = 12, sd = 9.4; Corsi m = 3.5, sd = 1.5; Digit SPAN m = 5, sd = 0.9; FAB m = 11.4, sd = 3.8; Fonemic Fluency m = 22.04, sd = 11; G8 m = 11.7, sd = 2.5; IADL m = 6.6, sd = 1.6; Prose Memory m = 8.29, sd = 4.8; Raven’s Matrices m = 21.2, sd = 13.2; Rey Pic Imm m = 27.16, sd = 10.9; Rey Pic Diff m = 9.18, sd = 7.3; Semantic Fluency m = 13.6, sd = 6.1; TMT-A m = 65.5, sd = 42.7; TMT-B m = 198.5, sd = 167). Olfactory psychophysical assessment, scored with the Sniffin’ Sticks Test, highlighted hyposmia (Sniffing m = 5.7, sd = 2.3; minimum score 2-, maximum score 8).

The one-way ANOVA did not show significant differences in psychophysical olfactory characteristics between genders, which were assessed through the Sniffin’ Sticks Test (F = 0.586; *p* = 0.448; η^2^ = 0.012).

Shapiro–Wilk normality tests were performed on each neuropsychological test to assess the sample’s normality. The following tests presented a nonparametric distribution: MMSE, TMT-A, TMT-B, BAI, ADL, IADL 15 Ray W Diff and 15 Ray W IMM, and Prose Memory. A single Kruskal–Wallis test was performed for each nonparametric distribution of the cognitive scales to analyze nonparametric scores.

**Figure 2:**
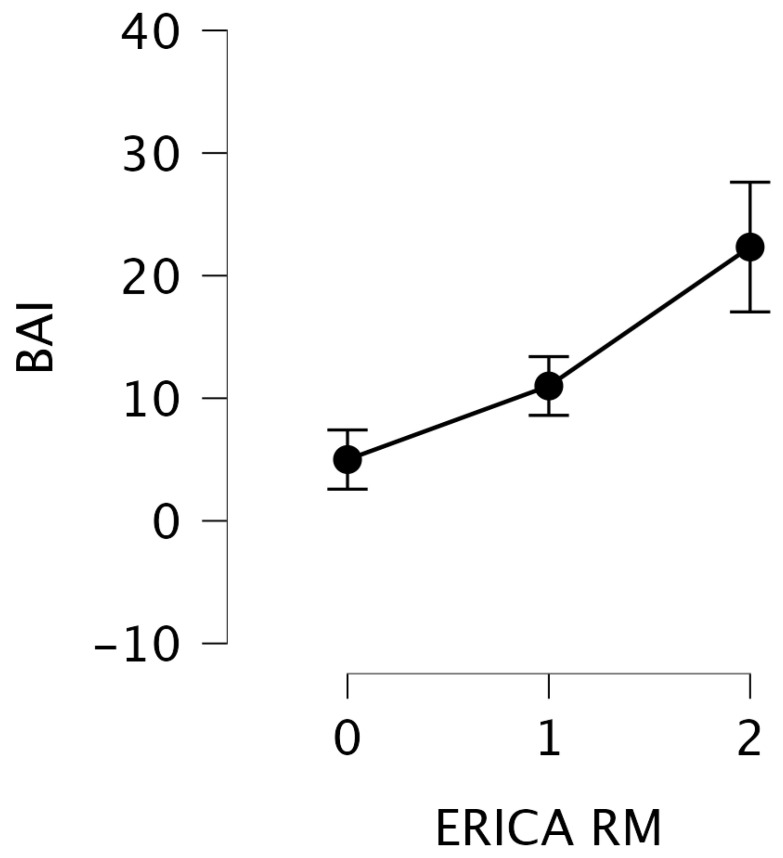
Graphical representation of BAI on the *y*-axis and ERICA RM on the *x*-axis. It can be observed that as BAI—which means the level of anxious symptoms—increases, there is a corresponding increase in the degree of atrophy in the perientorhinal/hippocampal cortex and the related ERICA RM score.

One-way nonparametric ANOVA highlighted significant differences only for the BAI test (Kruskal–Wallis test statistic 9.62, *p* = 0.008). Post hoc comparison showed significant differences between ERICA scores of 0 and 2 (t = −3.8, p_tukey_ = 0.004) and a result at the limit of significance between ERICA scores of 1 and 2 (t = −2.5, p_tukey_ = 0.057), in the direction of an increase in BAI linked to the increase in ERICA scores (0 = mean 5, sd = 4.30; 1 = mean 11, sd = 5.86; 2 = mean 22.33, sd = 12.95) (Figure 2). BAI values for ERICA score 3 were missing (6 patients). Other neuropsychological tests showed no significant differences (see Table 1 for nonparametric ANOVA and Table 2 for one-way ANOVA).

As the BAI increases, indicating a higher degree of anxiety symptomatology, the degree of atrophy in the perientorhinal/hippocampal cortex correspondingly increases, as reflected by a higher ERICA score (Figure 2).

**Figure 3:**
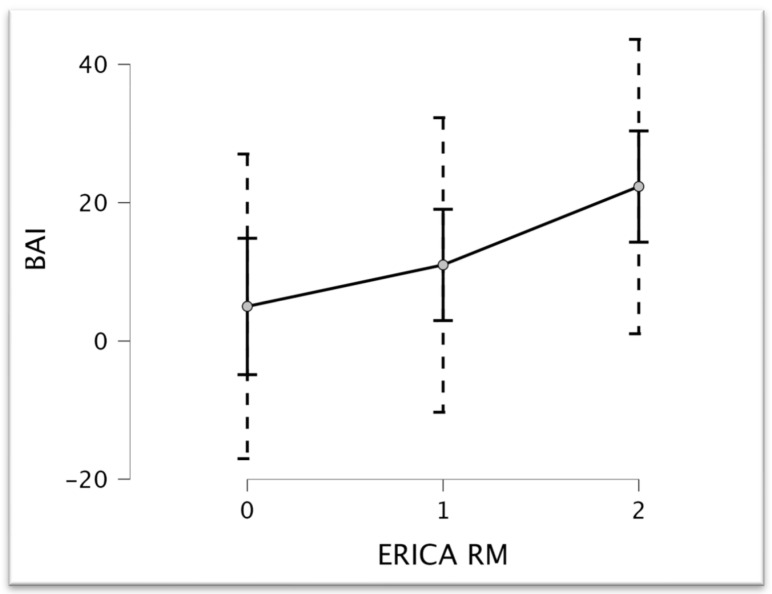
Plot of the marginal effect of ERICA RM on BAI scores.

Multiple regression analysis (STEPWISE method), considering ERICA as the dependent variable and neuropsychological tests as the covariate (R^2^ = 0.554, RMSE = 0.515, F = 16.125, *p* = 0.001), confirmed the ANOVA results. BAI scores are significant to the multiple regression model (t = 4.016, *p* = 0.001) (see Figure 3).

**Figure 4:**
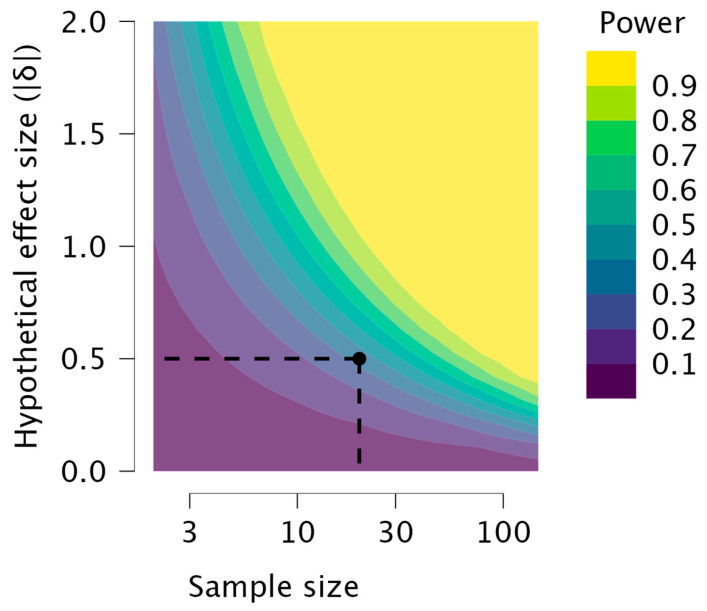
The power contour plot shows how the test’s sensitivity changes with the hypothetical effect size and the sample sizes in the design. As the sample sizes increase, smaller effect sizes become reliably detectable. The point shows the power of the specified design and effect size.

Power analysis (independent sample *t*-test), carried out after the study had already been completed, shows us the limits of our model’s power. The power contour plot (Figure 4) shows how the sensitivity of the test changes with the hypothetical effect size and the sample sizes in the design. As we increase the sample sizes, smaller effect sizes become reliably detectable. Conversely, smaller sample sizes are needed if one is satisfied by reliably detecting only larger effect sizes. The point shows the power of the specified design and effect size.

## 4. Discussion and Limits of the Study

This study examines the correlation between the degree of perientorhinal and hippocampal atrophy assessed by MRI and cognitive performance in a sample of hyposmic multidomain MCI patients, as evaluated through a comprehensive neuropsychological and psychophysical evaluation. Our results indicate a positive correlation between the degree of anxious symptomatology and the degree of atrophy of the perirhinal/hippocampal cortex, measured through the ERICA score. ERICA score [1] was used because several neuropathological, volumetric, and functional MRI studies have already shown that the entorhinal cortex and the transentorhinal region are among the first brain structures to show pathological changes in AD, even before such changes appear in the hippocampus [27]. One possible hypothesis to explain this observation is that anxiety may contribute to neurodegenerative processes by inducing chronic stress and inflammation [37]. Stress has been implicated in the pathogenesis of several neurodegenerative disorders, including Alzheimer’s disease, through its effects on the hypothalamic–pituitary–adrenal (HPA) axis [38] and the release of stress hormones such as cortisol [39]. Chronic stress can also lead to increased inflammation in the brain, which has been linked to the development and progression of neurodegenerative diseases [40]. Recent studies have indicated that anxiety can be an independent risk factor for Alzheimer’s disease, separate from the presence of depression. Anxiety is particularly prevalent among MCI patients compared to those with more advanced stages of dementia, especially if they retain insight into their condition. Much of the literature suggests that anxiety is a result of Alzheimer’s disease neuropathology rather than hippocampal damage from anxiety-induced mechanisms, such as elevated cortisol levels. Higher anxiety levels correlate with lower metabolism in the bilateral entorhinal cortex, bilateral anterior parahippocampal gyrus, left anterior superior temporal gyrus, and left insula [41]. Another possible explanation is that anxiety and depression may be early symptoms of neurodegeneration rather than risk factors for its progression [42]. This hypothesis is supported by previous studies that have shown a high prevalence of depression and anxiety in individuals with MCI, and by the fact that these symptoms often precede the onset of cognitive impairment in neurodegenerative diseases [43,44]. However, it is also important to note that the observed correlation between anxiety symptoms and cortical atrophy does not establish a causal relationship between the two—it is possible that other factors, such as genetic predisposition or environmental exposures, may be responsible for both anxiety symptoms and cortical atrophy [45,46]. Furthermore, the observed correlation may be confounded by factors, such as age or gender, not fully accounted for in this study. Thus, further research is needed in order to clarify the relationship between anxiety symptoms and cortical atrophy in MCI patients, and to explore the underlying mechanisms that may be involved. Longitudinal studies may be useful in determining whether anxiety symptoms predict the progression of cortical atrophy over time, or whether they are simply a marker of ongoing neurodegenerative processes. The findings of our study also have implications for the development of interventions to prevent or slow down the progression of MCI and dementia. These interventions may encompass a spectrum of approaches, including cognitive behavioral therapy for anxiety, stress management techniques, or other interventions aimed at reducing anxiety symptoms. In addition, incorporating the use of neuroimaging techniques into routine clinical practice to monitor brain atrophy in MCI patients with anxiety symptoms could help to identify those at higher risk of cognitive decline and inform targeted interventions. By identifying individuals at heightened risk of cognitive decline through such imaging assessments, clinicians can devise targeted intervention strategies to mitigate progression. This proactive approach not only facilitates early intervention, but also enables the customization of treatment plans to address the unique needs of each patient. In essence, leveraging both psychological interventions and neuroimaging technologies presents a promising avenue for optimizing clinical care and enhancing outcomes for individuals with MCI. By integrating these approaches into comprehensive care plans, healthcare providers can take proactive steps toward managing MCI more effectively and ultimately improve affected individuals’ quality of life.

Although our study provides critical preliminary findings, it is essential to acknowledge some of its limitations and provide a more comprehensive context for our findings. Firstly, the small sample size, the low power of the design, and the absence of a control group without MCI may limit the generalizability of our results. Moreover, with it being an exploratory study of many of the components involved, we were not able to take into consideration finer analyses that could discriminate the various types of anxiety, such as, for example, mixed anxiety and depressive disorder, the incipit of late-life depression, or generalized anxiety disorder. Furthermore, subjects with psychiatric disorders were excluded from the sample; therefore, MCI patients with full-blown depressive comorbidity who were undergoing pharmacological treatment for depression were not recruited. This did not allow us to evaluate an effective continuum on the overlap and evolution of anxiety and depression, as Beck’s model indicates [47]. Another critical limitation, precisely concerning the focus on anxiety, was the lack of analysis of the types of anxiolytics and antidepressants, if used, by the patients who had been recruited into the study. A further factor predisposing the establishment of a process of cognitive impairment could also be pharmacological, because some anxiolytics and antidepressants, taken chronically, could lead to AD [48]. Additionally, our study focused specifically on the entorhinal cortex and parahippocampal gyrus, but anxiety symptoms may also be related to atrophy in other brain regions. For example, different levels of atrophy could be present in the insula and right putamen, or the parahippocampal and hippocampal areas [48].

A further important limitation of the study is that it did not consider the aspect of SARS-CoV-2 viral infection—which, in other studies, we have considered and compared to MCI—given the possibility of etiopathogenetic overlap, especially for the part connected to the impairment of olfactory functionality (also evidenced by atrophy of the bulb) [49].

Last but not least, we did not perform a follow-up to check the evolution of the disease.

Therefore, future studies with larger sample sizes and comparison groups are warranted to confirm our findings. Additionally, our cross-sectional design precludes us from establishing causality between anxiety symptoms, cortical atrophy, and MCI. Longitudinal studies that follow patients over time are necessary to establish the temporal relationships between these variables and to determine the potential predictive value of neuropsychological scores and brain atrophy measures for MCI progression. Such longitudinal approaches will produce invaluable insights into the underlying mechanisms driving cognitive decline, and facilitate the development of more efficacious interventions and treatment modalities for individuals at risk of MCI.

Additionally, it is important to highlight, as we said before, that our study predominantly focuses on the entorhinal cortex and hippocampal gyrus—this selective emphasis may impact the interpretation of our findings, as other brain regions could also significantly influence the observed correlations. Consequently, future research endeavors should strive to include a more comprehensive assessment of these factors to better elucidate their contributions to the relationship between anxiety symptoms, cortical atrophy, and MCI. Despite these limitations, our study contributes to the growing body of literature investigating the possible relationship between anxiety symptoms and brain atrophy in MCI patients, highlighting the need for further investigation in this area.

## 5. Conclusions

In conclusion, the results of our study suggest that anxiety may play a critical role in the progression of MCI. This discovery sheds light on the intricate nature of MCI and highlights the need for a comprehensive assessment that considers neuroimaging techniques and neuropsychological evaluations. Integrating these two assessment tools may offer clinicians and researchers a deeper understanding of MCI, leading to more accurate diagnoses and targeted treatment options. By acknowledging the pivotal role of psychological factors, such as anxiety, in MCI progression, we stand to enhance the early detection and ongoing management of MCI patients. This proactive approach not only holds the potential for improving patient outcomes, but also contributes to enhancing their overall quality of life. As we unravel the multifaceted nature of MCI, integrating psychological considerations into clinical practice becomes increasingly imperative for delivering personalized and effective care to individuals affected by this condition.

## Figures and Tables

**Table 1:** The results of the nonparametric ANOVA (Kruskal–Wallis test) performed on nonparametric distribution of the cognitive scales: MMSE, TMT-A, TMT-B, BAI, ADL, IADL 15 Ray W Diff and 15 Ray W IMM, Prose Memory.

Test	Statistic	*p*
MMSE	2.6	0.46
TMT-A	2.05	0.56
TMT-B	0.81	0.84
BAI	9.62	0.008
ADL	7.24	0.06
IADL	2.77	0.43
15 Ray W IMM	1.29	0.73
15 Ray W Diff	3.09	0.38
Prose Memory	1.14	0.77

**Table 2:** The results of the one-way ANOVA, expressed in terms of the experimental values of the F tests and *p*-value. The analysis considered ERICA scores as fixed factors and neuropsychological assessments as dependent variables.

Test	F	*p*	*η* ^2^
BDI	0.651	0.542	0.115
Corsi	1.417	0.252	0.096
Digit Span	1.482	0.231	0.086
FAB	0.774	0.515	0.055
Fonemic Fluency	0.402	0.752	0.029
G8	0.049	0.737	0.536
Prose Memory	0.29	0.832	0.023
Raven’s Matrices	2.672	0.060	0.160
Rey’s Pic Diff	0.051	0.984	0.004
Rey’s Pic Imm	0.407	0.749	0.030
Semantic Fluency	0.051	0.984	0.004
Sniffing	1.323	0.279	0.081

## Data Availability

Data are available upon request from the corresponding author. The data are not publicly available as they contain information that could compromise the privacy of research participants.
